# The Febrifuge Action of Cold Water Used Internally

**Published:** 1870-08

**Authors:** James S. Bailey

**Affiliations:** Albany, N. Y.


ART II.—*The Febrifuge Action of Cold Water used internally.* By JAMES S. BAILEY, M. D., Albany, N. Y.

During the late war, while standing in a Southern Depot, I was attracted by the groans of a man lying upon the floor in the corner of the room apparently suffering much. It was evident that he had had a chill, closely approximating congestion, and now, that reaction was established, in the delirium of fever he was muttering incoherently. I handed him a pint cup full of water which he drank much to the relief of his parched throat; I gave him another, which seemed to slake his thirst; a third cup was handed him, and with a little persuasion he drank it also, but in a few moments all three were vomited. I then insisted upon his taking the fourth cupful, when he again vomited the water, with large quantities of dark ropy bile. He was soon bathed in a profuse prespiration, the circulation became equalized, and there was an abatement of fever. I then prescribed Sulph. Quinine grs. v, and Sulph. Morphia gr. 1-16; Pulv. Ipicac gr. 1-4—every three hours. By the next morning he was enabled to resume his duties.

Within the last month I was called to prescribe for a young lady (A Lousiana Creole) who had just arrived in this city from Aspinwall, after a residence of twelve months in that locality. She was suffering from an attack of miasmatic fever, and represented the anemic appearance so common among persons whose blood has been thined by a long residence in a warm climate. The spleen was much enlarged and congested, and consequently there was considerable tenderness and pain over this region. The nausea was successive, and she vomited considerable blood, with matter deeply tinged with bile; her pulse numbered 120 and was small and thready; the countenance wore a peculiar pinched expression; skin dry and hot, and thirst excessive. Such was her condition when I first saw her. I immediately prescribed the free use of cold water internally; when she had drank about three pints she vomited copiously, which produced relaxation of the system and she prespired freely. The temperature was soon reduced, and she passed into a quiet and refreshing sleep. By the use of autipeniodics and tonics she was soon sufficiently improved to resume her journey homeward.

I mention these cases to illustrate the febrifuge action of cold water. There is nothing in this stage of fever which acts so charmingly; it not only allays thirst, but unlocks the secretions, causing copious prespirations, reducing the temperature of the body, and equalizing the circulation in a degree which nothing else so effectually accomplishes.

The practice of causing a patient with fever to abstain from the use of water is cruel in the extreme, and very unnecessary; a full draught of cold water will not produce congestion of the stomach and bowels, but will produce the effects just described.

The absorbants in fever are not so active as in health, and the water in contact with the fevered surface of the stomach soon becomes heated and is rejected, which is followed by relaxation and a determination of the blood to the surface, which is a desirable result.

Cold water under proper restrictions is applicable to every stage and form of disease. If it acts, otherwise such cases are the exception, not the rule. Water as a therapeutic agent may be abused, every remedy is liable to misuse, but we cannot think that the thoughtful physician will attempt to ride this as a hobby to the exclusion of reason and common sense.

The physician accustomed to observe the workings of nature in health and disease, never fails to be admonished by the cravings of the system. The internal use of this remedy is just as necessary and satisfactory, as is its use externally in quieting and reducing the temperature of the system. The broad extended views of the educated physician are not hampered by isms and pathys, but he is at liberty to embrace and use all of nature's curative agents which are calculated to aid in the alleviation of suffering and disease.

